# Early risk stratification using Rubidium-82 positron emission tomography in STEMI patients

**DOI:** 10.1007/s12350-017-0993-x

**Published:** 2017-07-17

**Authors:** Adam Ali Ghotbi, Philip Hasbak, Lars Nepper-Christensen, Jacob Lønborg, Kiril Atharovski, Thomas Christensen, Lene Holmvang, Thomas Engstrøm, Rasmus Sejersten Ripa, Andreas Kjær

**Affiliations:** 1 Department of Clinical Physiology, Nuclear Medicine & PET and Cluster for Molecular Imaging, Rigshospitalet University of Copenhagen, Blegdamsvej 9, 2100 Copenhagen, Denmark; 2 Department of Cardiology, The Heart Center, Rigshospitalet University of Copenhagen, Copenhagen, Denmark

**Keywords:** Positron emission tomography, cardiac magnetic resonance, Rubidium-82, myocardial blood flow, ST-segment elevation myocardial infarction, final infarct size

## Abstract

**Background:**

Assessment of infarct size after myocardial infarction is predictive of subsequent morphological changes and clinical outcome. This study aimed to assess subacute post-intervention Rubidium-82 (^82^Rb)-PET imaging in predicting left ventricle ejection fraction, regional wall motion, and final infarct size by CMR at 3-months after STEMI.

**Methods:**

STEMI patients undergoing percutaneous coronary intervention were included prospectively. Rest-only ^82^Rb-PET perfusion imaging was performed at median 36 hours [IQR: 22 to 50] after the treatment. The extent of hypoperfusion and absolute blood flow (mL·min·g) were estimated on a global and a 17-segment model with dedicated software. At 3-months follow-up patients completed the CMR functional and late gadolinium enhancement imaging.

**Results:**

42 patients were included, but only 35 had follow-up CMR and constituted the study population. Absolute blood flow was significantly lower in the infarct-related territory compared to remote myocardium, *P* < .005. Extent of hypoperfusion correlated with final infarct size, *r* = 0.58, *P* < .001, while blood flow correlated with ejection fraction, *r* = 0.41, *P* < .05. In linear mixed models, higher subacute absolute blood flow (*β* = 4.6, confidence interval [3.5; 5.2], *P* < .001, *R*^2^ = 0.67) was associated with greater wall motion. Segmental extent of subacute hypoperfusion (*β* = 0.43 [0.38; 0.49], *P* < .001, *R*^2^ = 0.58) was associated with the degree of late gadolinium enhancement at 3-months.

**Conclusions:**

Subacute rest-only ^82^Rb-PET is feasible following STEMI and seems predictive of myocardial function and infarct size at 3-months.

**Electronic supplementary material:**

The online version of this article (doi:10.1007/s12350-017-0993-x) contains supplementary material, which is available to authorized users.

## Introduction

The assessment of left ventricular function and final infarct size (FIS) in the chronic phase after an ST-elevation myocardial infarction (STEMI) is clinically important because these parameters impact patient management and risk stratification.^1^–^3^ Infarct size measured by single-photon emission computed tomography (SPECT) at discharge after STEMI has been extensively validated and is predictive of subsequent morphological and functional left ventricular (LV) changes.^4^–^8^ Albeit readily available at most institutions, its use has not become clinical routine in prognostic evaluation of STEMI patients, perhaps due to low resolution and high time consumption. Another post-pPCI imaging method to risk-stratify STEMI patients is cardiac magnetic resonance (CMR). CMR-derived measurements have been associated with LV morphological changes and clinical outcome after STEMI.^3^,^9^,^10^ However, CMR cannot be performed in a considerable number of patients due to contraindications, and it is also a time-consuming technique. Rest positron emission tomography (PET) using Rubidium-82 (^82^Rb) tracer has no absolute contraindications and provides rest myocardial perfusion imaging (MPI) in less than 15 minutes in absolute terms (milliliters·minute·gram), which extends the scope of conventional semi-quantitative SPECT. The time aspect combined with the possibility of proper patient monitoring during the examination becomes increasingly important with the decreasing hospitalization time and the widespread use of early transfer (same day) to local hospitals that may not have the same imaging facilities. We have previously reported about the feasibility of ^82^Rb-PET in assessing the infarct size subacutely after STEMI compared to SPECT and CMR.^11^

This prospective cohort study aims to uncover whether PET derived measurements of resting blood flow and extent of severe hypoperfusion in the subacute phase of STEMI can predict final infarct size, LVEF, and regional wall motion as measured by CMR at 3-months follow-up.

## Methods

**Figure 1:**
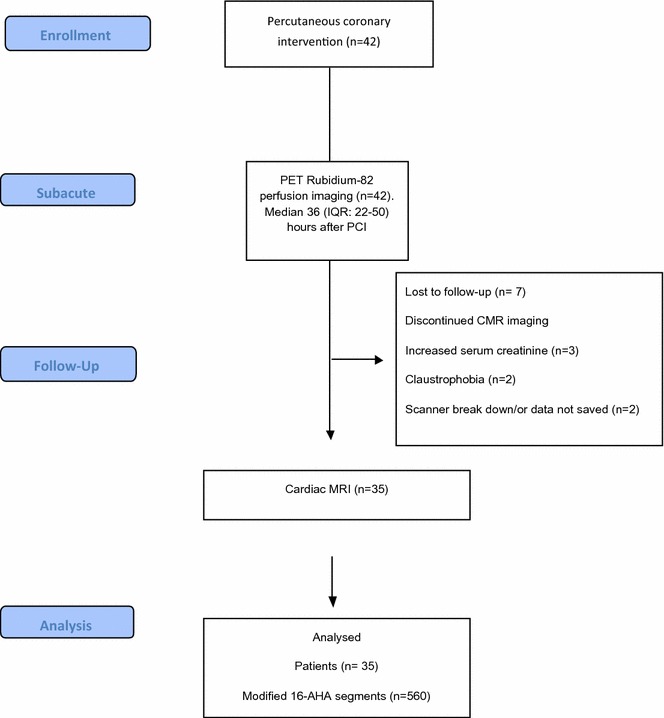
Study design. *PET*, positron emission tomography; *CMR*, cardiac magnetic resonance imaging; *AHA*, American Heart Association

Patients with index STEMI were prospectively included in the study (Figure 1). Other results from 12 of these patients have previously been reported in a separate study.^11^ Inclusion criteria were symptom duration less than 12 hours and STEMI in the ECG defined as ST- segment elevation in 2 contiguous electrocardiographic (ECG) leads of  >0.1 mV in V_4_ − V_6_ or leads II, III, and aVR, or >0.2 mV in lead V_1_ − V_3_. Exclusion criteria were cardiogenic shock, previous MI, stent thrombosis, unconsciousness, or previous coronary artery bypass grafting. All patients were pretreated with standard therapy, including oxygen, sublingual nitroglycerin, aspirin (300 mg), prasugrel (60 mg), and heparin (10,000 units i.v.) in the prehospital setting. The infarct-related vessel was identified by coronary angiography, and pPCI was performed according to international guidelines. Patients were treated lifelong with 75 mg aspirin and 10 mg prasugrel daily for 12 months.

Standard echocardiography was performed according to guidelines, routinely at discharge. Blood was sampled for creatine-kinase MB and Troponin T immediately before PCI and four additional times at fixed time-points post-PCI.

At 3-months follow-up, patients were interviewed to exclude any admission for restenosis/angina, and in addition, patient journals were audited.

### Ethics

The independent local ethics committee approved this study, protocol no: H-4-2010-054. All patients included received verbal and written information, and written consent was obtained from all the patients before inclusion in compliance with the Declaration of Helsinki.

### PET Acquisition

Rest PET MPI was performed the subsequent day after pPCI and when patients were clinically stable. The protocol has been described previously.^11^ In brief, patients were under constant visual and ECG monitoring and placed in supine position in a Siemens Biograph mCT/PET 64-slice scanner (Siemens Medical, Knoxville, TN, USA). For attenuation correction, a low-dose CT scan was acquired, and subsequently, patients were administrated approximately 1100 MBq of ^82^Rb i.v. (Cardiogen·82^®^, Bracco Diagnostics Inc., USA) and underwent 7 minutes of 3D list mode dynamic data acquisition. PET and CT images were manually inspected for any misalignment and corrected if necessary before reconstruction of attenuation-corrected images. Images were reconstructed into 21 frames (12 × 10 seconds, 3 × 20 seconds, 6 × 30 seconds) with attenuation, scatter, and decay corrections using 3D iterative ordered-subsets expectation maximization, Gaussian filtering with 10 mm full width at half maximum, including time-of-flight and point-spread-function. Examination time was approximately 15 minutes.

Cedars QPS/QGS® software (v. 2012, Cedars Sinai, USA) was used to process and analyze semi-quantitative and quantitative data in a semi-automated manner. Two experienced observers assessed the accuracy of slice-alignments in the ventricular planes and intervened if necessary (blinded to CMR data). Disagreements were solved by consensus. Tracer uptake was normalized to maximal uptake (index 100).

Myocardial perfusion was quantified using two methods: extent of hypoperfusion and absolute myocardial blood flow. Quantitation of the *extent* of severe hypoperfusion in the left ventricle was determined by an automated algorithm with a threshold of 2.5 standard deviations (SD) below normal counts in each pixel and estimated as percent of the LV volume and per AHA-17 segment model.^12^ The normal limit approach with cut-off 2.5 SD was prespecified in our protocol and was chosen since it has previously been used to estimate final infarct size in SPECT.^13^ However, the optimal cut-off value for follow-up assessments after STEMI remains unknown when using PET.^11^*Absolute* rest myocardial blood flow (ml·min·g) was calculated by applying Lortie’s one compartment model^14^ and was expressed as global myocardial blood flow, regional myocardial blood flow, and myocardial blood flow per AHA-17 segment. Rest myocardial blood flow was indexed to rate-pressure product and multiplied by 10,000 mmHg·s. The AHA-17 segment model was reformatted to a 16-segment model (the apical segment was disregarded) to accommodate the corresponding CMR 16-segment model.

### CMR Acquisition

Follow-up imaging was performed 3 months after pPCI on a 1.5 T scanner (Avanto, Siemens, Erlangen, Germany) with the use of a 6-channel body array coil. Functional assessment of the LV was performed using an ECG-triggered balanced steady-state free-precession cine sequence covering the entire LV (slice thickness 8 mm, slice gap 0 mm, echo time 1.5 ms, field of view 300 mm, and 25 frames per heart beat). Final infarct size (FIS) was quantified 10 minutes after administration of Gadolinium-based contrast (0.1 mL·kg; Gadovist, Bayer Schering, Berlin, Germany). An ECG-triggered enhancement inversion-recovery was used (slice thickness, 8 mm; field of view, 300 to 360 mm; echo time, 1.4 ms, slice gap 0 mm). In short-axis images of the LV, the inversion time was adjusted in each slice from base to apex in order to null the signal from the normal myocardium.

On short-axis cine images endo- and epicardial contours were manually drawn on End-diastolic and End-systolic frames. Papillary muscles were incorporated into the LV lumen volume. LV volumes, wall thickening in percent, and wall motion in millimeters were subsequently obtained automatically from the software.

Wall thickening was calculated by expressing the end-systolic increase for each AHA-16 segment as a percentage of its end-diastolic wall thickness ((end-systolic − end-diastolic) × 100/ end-diastolic). Each AHA-16 segment was categorized as normal, hypokinetic or hyperkinetic based on a normal database of wall thickening.^15^ Modified AHA-16 segments polar maps were generated after manually determining the right ventricle’s superior and inferior insertion on the LV.

FIS was assessed on the late gadolinium enhancement images after manual endo- and epicardial contour tracking. FIS was defined as hyper-intensive myocardium (>5 SD of the mean intensity of normal reference myocardium). FIS was calculated as percent of the LV volume and percent transmurality per AHA-16 segment. The analyses were performed by two experienced observers that were blinded to PET data using CVI^42^ (Circle Cardiovascular Imaging Inc., Calgary, Canada)

### Statistical Analyses

Continuous normally distributed variables are summarized as mean±SD and non-normally distributed continuous variables as median (interquartile range, [IQR]) and categorical variables as frequencies or percent (%). Generalized Estimating Equations’ test was used when comparing three variables. The Mann-Whitey test was used for comparisons between two independent groups. Correlation between any two modalities was examined with Spearman’s rank correlation. Correlations less than 0.39 were interpreted as “fair,” values between 0.40 and 0.59 as “moderate,” and values above as “strong”.^16^

In order to assess the predictive ability of subacute ^82^Rb-PET for follow-up cardiac function and morphology three different sets of predictors corresponding to models of increasing complexity were considered. Model 1 contains only PET derivatives as explanatory variables; model 2, in addition, included AHA-17 segments, infarct-related artery segments, and vascular territory. These variables were included to adjust for differences in regional tracer uptake due to physiological and pathophysiological circumstances^17^,^18^; model 3 further included age, gender, tobacco, diabetes, hypercholesterolemia, hypertension, time from symptom-onset to pPCI, apoplexia, familiar disposition, and Thrombolysis In Myocardial Infarction-flow, which are commonly acknowledged as risk factors in follow-up morbidity and mortality and infarct size after STEMI.^19^ Linear mixed models were used for the analyses in order to account for correlation between segments belonging to the same subjects. For comparison, mixed model analyses were also performed using routine discharge echocardiography ejection fraction; and peak Troponin T and peak creatine-kinase MB as predictors. For all predictive models, the *R*^*2*^ (variance explained) was estimated according to Nakagawa et al^20^ All statistical analyses were performed using SPSS® version 19 (IBM SPSS, Chicago, IL, USA).

## Results

A total of 42 Patients were recruited for the study, but seven were lost to follow-up CMR (Figure 1). The 35 patients with both subacute ^82^Rb-PET MPI and follow-up CMR constituted the study population. Baseline demographics and medical history for patients enrolled are summarized in Table 1. The culprit artery was left anterior descending artery in 53% of the patients, and 69% had a Thrombolysis In Myocardial Infarction-flow of 0 to 1 prior to pPCI.

**Table 1:** Baseline characteristics

	Patients (*n* = 35)
Age	58 ± 9 years
Male	29 (83%)
Hypertension	11 (26%)
Hypercholesterolemia	7 (17%)
Total cholesterol, mmol/L	4.9±0.8 mmol/L
Diabetes	1 (2%)
Smoking	
Non	10 (24%)
Active	18 (43%)
Ex	14 (33%)
Family history of premature CAD	17 (41%)
Peripheral arterial disease	0
Infarct location	
LAD	17 (49%)
RCA	15 (42%)
LCX	2 (6%)
LM	1 (3%)
TIMI flow prior to pPCI	
0	17 (49%)
1	8 (23%)
2	5 (14%)
3	5 (14%)
TIMI flow post-pPCI	
0	1 (2%)
1	0
2	3 (9%)
3	31 (89%)
Peak troponin T, ng/mL	5769 ± 6201
Peak CK-MB, U/I	247 ± 175
Ejection fraction post-pPCI at discharge, %	43.8 ± 9.6
Time from symptom-onset to PCI, minutes (IQR)	165 (120–273)
Medication during follow-up	
ACE/ARB	11 (31%)
Beta-blockers	31 (88%)
Statins	35 (100%)
Thiazide/loop diuretic	6 (17%)
Acetylsalicylic acid	35 (100%)
ADP antagonist	35 (100%)

*CAD*, coronary artery disease; *LAD,* left anterior ascending artery; *RCA*, right coronary artery; *LCX*, left circumflex artery; *LM*, left main artery; *TIMI*, thrombolysis in myocardial infarction; *pPCI*, post-primary percutaneous intervention; *CK-MB*, Creatine-kinase MB; *IQR*, interquartile range; *ACE/ARB*, angiotensin-converting-enzyme inhibitor/angiotensin receptor II blocker; *ADP antagonist,* adenosine diphosphate (ADP) receptor inhibitor

### PET Measurements

Rest PET MPI was performed at a median of 36 hours [22; 50] after pPCI. The median myocardial area with severe hypoperfusion was 20.0% of the LV [IQR: 7.5; 35.0] in the subacute phase. Absolute median global blood flow, blood flow in the infarct-related artery territory (IRA), and blood flow in the non-IRA territory were 0.94 [0.78; 1.12], 0.71 [0.57; 0.95] and 1.0 [0.86; 1.16] mL·min·g, respectively. Blood flow at IRA territory was significantly lower than the global blood flow and the blood flow at non-IRA territory (*P* < .005 for difference).

### CMR Measurements

At follow-up (median 93 days, [91; 96] 35 patients were examined. Median LVEF was 55% [49; 60]. Median LV End-diastolic and End-systolic volumes were 172 mL [143; 210] and 79 mL [57; 98], respectively. Median FIS was 13.9% [9.5; 22.0] of the LV volume. A total of 273 out of 560 segments (48%) had late gadolinium enhancement (LGE; transmurality range 1 to 97%, median 17% [3; 41]). LGE, wall thickening, and wall motion stratified by wall function categories are listed in Table 2.

**Table 2:** Follow-up CMR measurements of wall thickening and subacute blood flow by ^82^Rb-PET on segmental level

	Hypokinetic	Normal	Hyperkinetic	*P* value
Segments (*n*)	238	263	59	
Wall thickening (%)	31 (11–45)*^,¶^	70 (57–86)	118 (108–128)	<.001
Wall motion (mm)	4.7 (2.4–7.1) *^,¶^	8.1 (5.9–10.6)	10.3 (7.9–12.3)	<.001
LGE follow-up, % of segment	13.8 (0–45.7)*^,¶^	0 (0–10.0)	0 (0–1.9)	<.001
^82^Rb—extent of severe hypoperfusion (% of segment)	13 (0–72)*^,¶^	0 (0-23)	0 (0–11)	<.001
^82^Rb—absolute blood flow (ml·min·g)	0.81 (0.63–1.02)*^,¶^	0.97 (0.78–1.20)	1.22 (0.93–1.34)^*^	<.001

Measurements from follow-up wall thickening and wall motion with CMR and subacute setting PET-Rb. Values are median (IQR). AHA-16 model segments of abnormal, normal, hyperkinetic wall thickening as measured by CMR and stratified according to normal database

*LGE follow-up*, late gadolinium enhancement at follow-up per segment; *Rb*, Rubidium-82 median (IQR) percent of extent severe hypoperfusion per segment, absolute blood flow in ml·min·g

^*^P < .001 for difference vs. normal wall thickening segments

^¶^*P* < .02 for difference vs. hyperkinetic wall thickening segments

### Relationship Between Subacute PET and Follow-Up CMR Measurements: Global Level

As presented in Table 3, PET derived variables correlated from “fair” to “moderate” with CMR derivatives. On a patient-to-patient basis, the best and strongest correlation was found between extent of severe hypoperfusion with CMR FIS. The extent of severe hypoperfusion had a moderate correlation with CMR LVEF and LV end-systolic volume at follow-up. Global absolute flow by PET had a moderate correlation with CMR LVEF and LV end-systolic volume at follow-up.

**Table 3:** Correlation between subacute PET and follow-up CMR

	Correlation coefficient (*r*)			
	CMR follow-up (*N* = 35)			
Variables	LVEF (%)	LVEDV (mL)	LVESV (mL)	FIS (% of LV)
Extent of severe hypoperfusion (%)	−0.53**	0.31	0.48**	0.58***
Global blood flow (ml·min·g)	0.41*	−0.22	−0.42*	−0.32

Correlations between PET derivatives and CMR follow-up outcomes on a patient basis

*CMR*, cardiac magnetic resonance imaging; *LVEF* left ventricle ejection fraction; *LVEDV* left ventricle end-diastolic volume; *LVESV* left ventricle end-systolic volume; *FIS* final infarct size; *PET*, positron emission tomography

* *P* < .05

** *P* < .01

*** *P* < .001

### Predictive Power of PET Measurements On a Segmental Level

The extent of hypoperfusion and myocardial blood flow stratified by wall function categories is summarized in Table 2.

In order to assess the predictive ability of subacute ^82^Rb-PET for follow-up cardiac function and morphology three different prediction models of increasing complexity were considered. Results are listed in Table 4 for each model with different adjusted variables. We found that model 2, which further includes AHA-17 segments, infarct-related artery segments, and vascular territory as predictors, best predicted the outcomes in terms of a substantially higher variances explained (i.e., *R*^2^) compared to the simple model which only includes the PET derivatives as predictors (model 1).^20^ Model 3 performed marginally better than model 2 in terms of *R*^2^, but at the expense of higher complexity. The estimates of model 2 are reported below.

**Table 4:** Linear mixed models for CMR derivatives explained by PET measurements

Model 1	CMR follow-up								
	No explanatory factors except PET derivatives *N* = 560 segments								
Outcome	Wall thickening (% AHA-16 segments)			Wall motion (mm AHA-16 segments)			LGE (% transmurality per AHA-16 segments)		
PET subacute	*β*	*P*	*R* ^2^	*β*	*P*	*R* ^2^	*β*	*P*	R^2^
Extent of severe hypoperfusion (%)	−0.28 [−.38; −.19]	.001	0.07	−0.06 [−.07; −.05]	.001	0.25	0.49 [.44; .53]	.001	0.48
Blood flow (ml·min·g)	62.2 [53.1; 71.3]	.001	0.30	6.0 [5.0; 7.0]	.001	0.25	−38.6 [−44.6; −32.6]	.001	0.28

Model 2	CMR follow-up								
	Controlled for: AHA-16 segments, infarct-related artery segments, vascular territory								
Outcome	Wall thickening (% AHA-16 segments)			Wall motion (mm AHA-16 segments)			LGE (% transmurality per AHA-16 segments)		
PET subacute	*β* [CI]	*P*	*R* ^2^	*β*	*P*	*R* ^2^	*β*	*P*	*R* ^2^
Extent of severe hypoperfusion (%)	−0.36 [−.45; −.27]	.001	0.37	−0.04 [−.05; −.03]	.001	0.66	0.43 [.38; .49]	.001	0.58
Blood flow (ml·min·g)	39.6 [28.5; 50.3]	.001	0.39	4.6 [3.5; 5.2]	.001	0.67	−38.4 [−44.8; −31.9]	.001	0.45

Model 3	CMR follow-up								
	Controlled for: AHA-16 segments, sex, vascular territory, infarct-related artery segments, HT, HL, DM, familiar disposition, apoplexia, tobacco, TIMI flow, time from symptom-onset to pPCI								
Outcome	Wall thickening (% AHA-16 segments)			Wall motion (mm) AHA-16 segments			LGE (% transmurality per AHA-16 segments)		
PET subacute	*β* [CI]	*P*	*R* ^2^	*β*	*P*	*R* ^2^	*β*	*P*	*R* ^2^
Extent of severe hypoperfusi-on (%)	−0.37 [−.47; −.26]	.001	0.42	−0.04 [−.05; −.03]	.001	0.68	0.42 [.37; .48]	.001	0.61
Blood flow (ml·min·g)	38.8 [26.8; 50.8]	.001	0.46	4.5 [3.6; 5.5]	.001	0.65	−38.6 [−45.7; −31.6]	.001	0.49

Mixed-model analyses (model 1, 2, and 3 with increasing complexity due to additional predictive factors and covariates) of CMR-derived wall thickening, wall motion and LGE transmurality per AHA-16 segments

*CI*, confidence interval; *PET*, positron emission tomography; *LGE*, Late gadolinium enhancement; *AHA-16,* American Heart Association 16-segment model

PET derived variables on a segment-to-segment basis were all significant predictors of CMR parameters. Absolute blood flow showed the best model fit for predicting wall motion at follow-up, (*β* = 4.6, confidence interval [3.5; 5.2], *P* < .001, *R*^2^ = 0.67); such that for every 0.1 mL·min·g increase of blood flow, there was a 0.46 mm increase in the wall motion during systole. The extent of severe hypoperfusion was best in predicting LGE transmurality, (*β* = 0.43 [0.38; 0.49], *P* < .001, *R*^2^ = 0.58); such that for every 1% increase of extent of severe hypoperfusion, there was 0.43% increase in LGE transmurality per segment.

Routine procedures such as echocardiography ejection fraction at discharge and blood samples of Troponin T and creatine-kinase MB are cheap and readily available tests. Therefore, a head-to-head comparison of new diagnostic tests is warranted. Variances explained by these tests were all substantially less than those explained by PET derivatives regarding CMR outcomes. See Table 5.

**Table 5:** Linear mixed models for CMR derivatives explained by EF at discharge, peak TNT and CK-MB

Outcome	CMR follow-up (*N* = 560 segments)								
	Wall thickening (% AHA-16 segments)			Wall motion (mm AHA-16 segments)			LGE (% transmurality per AHA-16 segments)		
	*β*	*P*	*R* ^2^	*β*	*P*	*R* ^2^	*β*	*P*	*R* ^2^
EF at discharge by Echo	0.99 [.38; 1.60]	.002	0.07	0.08 [.02; .13]	.008	0.002	−0.73 [−1.03; −.44]	.001	0.09
Peak TNT	−0.002 [−.003; −.001]	.001	0.07	−0.0002 [−.0003; −.00006]	.003	0.04	0.0013 [.0007; .002]	.001	0.06
Peak CK-MB	−0.06 [−.09; −.03]	.001	0.08	−0.005 [−.008; −.002]	.001	0.05	0.04 [.03; .06]	.001	0.12

Mixed-model regression analyses for routine tests readily available at cardiac units of CMR-derived wall thickening, wall motion, and LGE transmurality per AHA-16 segments

*EF*, ejection fraction; *Echo*, echocardiography; *TNT* troponin T; *CK-MB* creatine-kinase MB; *LGE*, late gadolinium enhancement; *AHA-16*, American Heart Association 16-segment model

### PET Measures of Extent of Severe Hypoperfusion and Blood Flow: Relation to CMR LVEF

**Figure 2:**
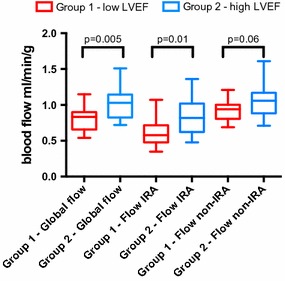
Blood flow measurements in low and high LVEF groups. *LVEF*, left ventricle ejection fraction; *IRA*, infarct-related artery

We stratified the cohort (*n* = 35) according to follow-up LVEF tertiles and compared patients in the lower third tertile (Group 1; *n* = 12) with the upper two tertiles (Group 2; *n* = 23). This was done in order to put the PET measurements at the subacute phase in the context of an important and valuable clinical parameter, LVEF. Median LVEF was 48.0% (43.0; 50.5) in Group 1 compared to 60% (55.0; 63.6) in Group 2 (*P* = .001). Baseline characteristics for the two groups are shown in Table 6. Median extent of severe hypoperfusion at the subacute phase was significantly higher in Group 1 (37%, [13; 46] compared to Group 2 (20%, [7; 27] (*P* = .02). Furthermore, global blood flow and blood flow in the IRA were significantly lower in Group 1 compared to Group 2, see Figure 2.

**Table 6:** Baseline characteristics of the study population grouped by lower and upper 2 tertiles of ejection fraction

	Group 1—low LVEF (*n* = 12)	Group 2—high LVEF (*n* = 23)	*P* value
Age, years (IQR)	51 (47;65)	60 (55;69)	.24
Systolic BP (mmHg)	129 (116;145)	123 (114;143)	.9
Diastolic BP (mmHg)	83 (68;94)	72 (64;82)	.09
Heart rate (/min)	68 (63;72)	61 (53;65)	.006
Coronary risk factors, *n* (%)			
Hypertension	4/12 (33)	7/23 (30)	.86
Smoking	2/12 (17)	12/23 (52)	.06
Diabetes mellitus	1/12 (8)	0/23 (0)	.16
Hyperlipidemia	2/12 (17)	3/23 (13)	.77
Apoplexia	0/12 (0)	1/23 (4)	.46
Peripheral arterial disease	0/12 (0)	0/23 (0)	
Familiar disposition	9/12 (75)	7/23 (30)	.009
PCI			
Time from symptom-onset to PCI, minutes	210 (120;330)	175 (122;267)	.99
Infarct location			
LAD	8 (53%)	11 (53%)	
RCA	3 (41%)	10 (41%)	.18
LCX	0 (0%)	2 (0%)	
LM	1 (2%)	0 (0%)	
TIMI flow before PCI			.89
0	6/12 (50)	12/23 (53)	
I	2/12 (17)	5/23 (21)	
II	2/12 (17)	3/23 (13)	
III	2/12 (17)	3/23 (13)	
TIMI flow after PCI			.39
0	0/12 (0)	1/23 (4)	
I	0/12 (0)	0/23 (0)	
II	2/12 (17)	1/23 (4)	
III	10/12 (83)	21/23 (91)	
Troponin T peak (ng/L)	5850 (5570;15200)	3570 (1855;5920)	.002
Creatine-kinase MB peak (ng/L)	320 (250;393)	157 (91;289)	.04

Values are given in median (*IQR*), interquartile range; *BP*, blood pressure; *PCI* percutaneous coronary intervention; *LAD,* left anterior descending artery; *RCA*, right coronary artery; *LCX*, left circumflex artery; *LM*, left main artery; *TIMI*, thrombolysis in myocardial infarction

## Discussion

The present study examined the ability of resting ^82^Rb-PET imaging performed in patients’ days after STEMI treated with pPCI to predict subsequent morphological features as detected with CMR at follow-up. We found in a small sample of STEMI population that PET MPI seems to forecast the degree of wall motion impairment and amount of scar. Furthermore, subacute blood flow measurement by PET was significantly lower in patients with reduced LVEF at 3-months follow-up compared to those with preserved LVEF. Together, these results are encouraging for further and larger trials investigating the clinical utility of ^82^Rb-PET in the subacute management of the STEMI patient for optimal and individualized risk stratification and tertiary prevention.

Gallagher et al established that an akinetic myocardial segment can either be infarcted, hibernating or stunned, and for the two latter conditions still salvageable.^21^ Hence, review of cardiac function after STEMI alone can conceal later functional recovery. Our study confirms that injury of the microcirculation as evaluated with ^82^Rb-PET after pPCI is associated with cardiac function at follow-up. This is important since the efficiency of pPCI and adjuvant therapy can be evaluated with ^82^Rb-PET in a fast, standardized and observer-independent way quickly after the STEMI.

Recently, perfusable tissue index (a marker for viability) derived from [^15^O]-H_2_O PET imaging, was shown to predict the recovery of segments assessed by CMR.^22^ This finding is in line with our results, where extent of severe hypoperfusion was predictive of the LGE transmurality at follow-up. ^82^Rb-PET imaging may be considered more advantageous with no need of an on-site cyclotron, which is necessary for producing [^15^O]-H_2_O. The monthly costs associated with an ^82^Rb-generator must be taken into consideration. But, since many centers use ^82^Rb MPI to assess the severity of coronary artery disease,^23^ it would be economically feasible to expand its role to the early post-STEMI phase once the procurement is established.

CMR-assessed FIS has previously been shown to be a strong predictor of subsequent cardiac events^3^,^10^ and the extent of LGE (segmental scar burden) has been shown to correspond to the histologically verified scar area.^24^ Therefore, the association between the extent of severe hypoperfusion with PET imaging and FIS and LGE transmurality derived from CMR at follow-up is an important aspect of this study. It emphasizes the potential of diagnostic and therapeutic targets in the microcirculation in patients following AMI.

Previously, research groups have demonstrated the benefits of myocardial perfusion imaging in post-infarction patients regarding functional recovery, LVEF, and cardiovascular death.^1^,^4^,^7^,^8^,^25^,^26^ Common for them are the use of conventional SPECT imaging which has lower spatial and temporal resolution than PET and is more cumbersome to perform,^23^ as well as mixed treatment strategies for STEMI (e.g., intravenous thrombolysis or PCI) and inconsistent treatment after STEMI.

A Mayo Clinic study demonstrated that discharge infarct size measured with ^99m^Tc- sestamibi on SPECT was not significantly correlated to discharge LVEF (determined by electron beam CT), but became so after remodeling had progressed at 6-weeks (*r* = −0.83, *P* = 0.0003) and 1-year (*r* = −0.84, *P* = 0.0002).^4^ The ^99m^Tc- sestamibi SPECT imaging was performed on average 7 ± 2 days after admission, whereas, our PET imaging was performed earlier at a median of 36 hours after primary PCI. This could be one explanation for the difference in correlation coefficient between ours and the Mayo Clinic study. Ndrepepa et al reported similar moderate correlation as ours between initial perfusion defect size and LVEF at follow-up examined in 626 patients with MI (*r* = −0.52, *P* < .001).^26^ The authors explained the relative moderate correlation as a result of a mismatch between ^99m^ Tc-sestamibi uptake and LVEF due to stunning. The extent of ^82^Rb uptake affected by stunning remains unclear. ^82^Rb is dependent on both the blood flow rate and the metabolic state of the myocardium.^27^ One preclinical study showed significantly lower absolute blood flow in areas of stunned myocardium compared to remote areas measured with ^82^Rb.

A study comparable to ours, confirm that areas with reduced wall motion were associated with decreased stress blood flow as well as reserve blood flow capacity as assessed 24 months after index MI with ^13^N-ammonia PET imaging.^28^ Similarly, Juárez-Orzco et al found that myocardial flow reserve in the spared myocardium in patients with previous MI was a better predictor of LVEF than infarct size.^29^ Our results suggest that areas with hypokinetic, normal, and hyperkinetic wall functioning at follow-up have dissimilar rest ^82^Rb-uptake already at the subacute state. Whether this is due to decreased extraction of the tracer during reactive hyperemia and/or the metabolic state of the myocardium as a ramification of the ischemia/reperfusion injury remains uncertain. We find that rest-only PET perfusion imaging in the subacute phase after MI seems sufficient to predict future cardiac contractility. However, it is well-known that the myocardial flow reserve adds more information regarding the microcirculatory status than rest-only imaging. We can only speculate if this would also be the case in our population. However, since stress testing is contraindicated in the subacute phase of an MI, this could not be tested in our study.

Our results could indicate that a single non-stress examination lasting less than 15 minutes performed during initial admission after an STEMI could add prognostic information to the cardiologist that could potentially individualize risk-management of follow-up surveillance. However, the ^82^Rb-PET examination has not been optimized for this indication. One example is our assessment of extent of hypoperfusion using a cut-off value of 2.5 SD, this value is derived from SPECT examinations of perfusion, and we have recently shown that this value is probably not optimal.^11^ SPECT and MRI are alternative methods that could be performed to gain similar information. These methods are more validated for this indication, but patient characteristics and/or local conditions may encounter when choosing the best method for the individual patient.

Another PET imaging method to predict regional and global LV function recovery is fluorodeoxyglucose (FDG). FDG-PET showed good sensitivity and specificity in predicting improvement in regional function after revascularization in patients with chronic coronary artery disease.^30^ FDG-imaging is a well-validated method for viability testing,^30^ but the interpretation of increased glucose uptake in the early stages after infarction is less clear 31 and could be influenced by the active myocardial inflammation.

Although the LVEF of group 1 (48%) was not substantially reduced and would be considered near normal, the ^82^Rb-PET imaging was able to differentiate between patients with high and low LVEF. Cuculi et al found no difference between resting and hyperemia flow with an invasive measuring technique at pPCI. This was probably due to microcirculatory jeopardization and therefore the inability to obtain hyperemia. But importantly, they found a significantly lower coronary flow reserve the following *day* in patients with lower LVEF compared to patients with preserved LVEF.^32^ Hence, the microcirculatory function changes during the first 24 hours after STEMI and is still affected although under recovery, stressing that the timing of MPI at approximately 24 hours after STEMI is feasible.

### Limitations

Of potential limitation is the lack of follow-up angiography to insure patent IRA. A silent reocclusion of the IRA after the reperfusion would lead to a deteriorating wall motion function. However, no patients were admitted to the hospital for AMI in the follow-up period, and no patients reported crescendo angina at the 3-months follow-up. Furthermore, the sample size is relatively small, and all participants were presented to a single center. Hence, confounders such as demographics and treatment can influence the results.

Potential mismatch between CMR and PET could be due to misalignment of segments. However, the standard use of AHA-16 segments should minimize this limitation. Finally, the optimal timing of ^82^Rb-PET is uncertain, and since it measures flow at a single time point, the condition of the microcirculation is uncertain as well.

## Conclusion

^82^Rb-PET imaging is feasible and can provide fast assessment of the absolute blood flow at the infarct territory and the extent of hypoperfusion following STEMI. These parameters seem predictive of myocardial function and infarct size at follow-up, factors that are of clinical importance for prognosis and morbidity.

The management of patients after infarction could thus potentially be enhanced with the knowledge provided by cardiac ^82^Rb-PET imaging and warrants further research in larger study populations.

## New Knowledge Gained

Our findings could expand the role of ^82^Rb-PET MPI in the early phase after STEMI and enable assessment of absolute blood flow to predict follow-up LV morphology and function in a time-efficient manner.

## Electronic supplementary material

Below is the link to the electronic supplementary material.

Supplementary material 1 (PPTX 1535 kb)

## Abbreviations

AHA-17: American heart association 17-segment model
AMI: Acute myocardial infarction
CMR: Cardiac magnetic resonance
FIS: Final infarct size
IRA: Infarct-related artery
LGE: Late gadolinium enhancement
LV: Left ventricle
LVEF: Left ventricle ejection fraction
MPI: Myocardial perfusion imaging
pPCI: Primary percutaneous coronary intervention
PET: Positron emission tomography
SPECT: Single-photon emission computed tomography
STEMI: ST-segment elevation myocardial infarction
^82^Rb: Rubidium-82
^99m^Tc: Technetium-99m

## References

[1] Gibbons RJ (2011). Tc-99m SPECT sestamibi for the measurement of infarct size. J Cardiovasc Pharmacol Ther.

[2] Korup E, Kober L, Torp-Pedersen C, Toft E (1999). Prognostic usefulness of repeated echocardiographic evaluation after acute myocardial infarction. TRACE Study Group. TRAndolapril Cardiac Evaluation. Am J Cardiol.

[3] Lonborg J, Vejlstrup N, Kelbaek H, Holmvang L, Jorgensen E, Helqvist S (2013). Final infarct size measured by cardiovascular magnetic resonance in patients with ST-elevation myocardial infarction predicts long-term clinical outcome: an observational study. Eur Heart J Cardiovasc Imaging.

[4] Chareonthaitawee P, Christian TF, Hirose K, Gibbons RJ, Rumberger JA (1995). Relation of initial infarct size to extent of left ventricular remodeling in the year after acute myocardial infarction. J Am Coll Cardiol.

[5] Christian TF, Gitter MJ, Miller TD, Gibbons RJ (1997). Prospective identification of myocardial stunning using technetium-99m sestamibi-based measurements of infarct size. J Am Coll Cardiol.

[6] Guenancia C, Cochet A, Humbert O, Dygai-Cochet I, Lorgis L, Zeller M (2013). Predictors of post-stress LVEF drop 6 months after reperfused myocardial infarction: a gated myocardial perfusion SPECT study. Ann Nucl Med.

[7] Sciagra R, Imperiale A, Antoniucci D, Migliorini A, Parodi G, Comis G (2004). Relationship of infarct size and severity versus left ventricular ejection fraction and volumes obtained from 99mTc-sestamibi gated single-photon emission computed tomography in patients treated with primary percutaneous coronary intervention. Eur J Nucl Med Mol Imaging.

[8] Miller TD, Christian TF, Hopfenspirger MR, Hodge DO, Gersh BJ, Gibbons RJ (1995). Infarct size after acute myocardial infarction measured by quantitative tomographic 99mTc sestamibi imaging predicts subsequent mortality. Circulation.

[9] Orn S, Manhenke C, Anand IS, Squire I, Nagel E, Edvardsen T (2007). Effect of left ventricular scar size, location, and transmurality on left ventricular remodeling with healed myocardial infarction. Am J Cardiol.

[10] El Aidi H, Adams A, Moons KG, Den Ruijter HM, Mali WP, Doevendans PA (2014). Cardiac magnetic resonance imaging findings and the risk of cardiovascular events in patients with recent myocardial infarction or suspected or known coronary artery disease: a systematic review of prognostic studies. J Am Coll Cardiol.

[11] Ghotbi AA, Kjaer A, Nepper-Christensen L, Ahtarovski KA, Lonborg JT, Vejlstrup N (2016). Subacute cardiac rubidium-82 positron emission tomography (82Rb-PET) to assess myocardial area at risk, final infarct size, and myocardial salvage after STEMI. J Nucl Cardiol.

[12] Cerqueira MD (2002). Standardized myocardial segmentation and nomenclature for tomographic imaging of the heart: A statement for healthcare professionals from the Cardiac Imaging Committee of the Council on Clinical Cardiology of the American Heart Association. Circulation.

[13] Fakhri Y, Busk M, Schoos MM, Terkelsen CJ, Kristensen SD, Wagner GS (2016). Evaluation of acute ischemia in pre-procedure ECG predicts myocardial salvage after primary PCI in STEMI patients with symptoms >12 hours. J Electrocardiol.

[14] Lortie M, Beanlands RSB, Yoshinaga K, Klein R, Dasilva JN (2007). DeKemp Ra. Quantification of myocardial blood flow with 82Rb dynamic PET imaging. Euro J Nucl Med Mol Imaging.

[15] Dawson DK, Maceira AM, Raj VJ, Graham C, Pennell DJ, Kilner PJ (2011). Regional thicknesses and thickening of compacted and trabeculated myocardial layers of the normal left ventricle studied by cardiovascular magnetic resonance. Circulation Cardiovasc Imaging.

[16] Altman DG (1991). Practical statistics for medical research.

[17] Hoffman JI (1995). Heterogeneity of myocardial blood flow. Basic Res Cardiol.

[18] Javadi MS, Lautamaki R, Merrill J, Voicu C, Epley W, McBride G (2010). Definition of vascular territories on myocardial perfusion images by integration with true coronary anatomy: A hybrid PET/CT analysis. J Nucl Med.

[19] Stone GW, Selker HP, Thiele H, Patel MR, Udelson JE, Ohman EM (2016). Relationship between infarct size and outcomes following primary PCI: Patient-level analysis from 10 randomized trials. J Am Coll Cardiol.

[20] Nakagawa S, Schielzeth H (2013). A general and simple method for obtaining R^2 from generalized linear mixed-effects models. Methods Ecol Evol.

[21] Gallagher KP, Kumada T, Koziol JA, McKown MD, Kemper WS, Ross J (1980). Significance of regional wall thickening abnormalities relative to transmural myocardial perfusion in anesthetized dogs. Circulation.

[22] Timmer SA, Teunissen PF, Danad I, Robbers LF, Raijmakers PG, Nijveldt R (2016). In vivo assessment of myocardial viability after acute myocardial infarction: A head-to-head comparison of the perfusable tissue index by PET and delayed contrast-enhanced CMR. J Nucl Cardiol.

[23] deKemp RA, Klein R, Beanlands RSB (2016). 82Rb PET imaging of myocardial blood flow—have we achieved the 4 “R”s to support routine use?. EJNMMI Res.

[24] Kim RJ, Fieno DS, Parrish TB, Harris K, Chen EL, Simonetti O (1999). Relationship of MRI delayed contrast enhancement to irreversible injury, infarct age, and contractile function. Circulation.

[25] Sciagra R, Bolognese L, Rovai D, Sestini S, Santoro GM, Cerisano G (1999). Detecting myocardial salvage after primary PTCA: early myocardial contrast echocardiography versus delayed sestamibi perfusion imaging. J Nucl Med.

[26] Ndrepepa G, Mehilli J, Martinoff S, Schwaiger M, Schomig A, Kastrati A (2007). Evolution of left ventricular ejection fraction and its relationship to infarct size after acute myocardial infarction. J Am Coll Cardiol.

[27] Lekx KS, deKemp RA, Beanlands RS, Wisenberg G, Wells RG, Stodilka RZ (2010). Quantification of regional myocardial blood flow in a canine model of stunned and infarcted myocardium: Comparison of rubidium-82 positron emission tomography with microspheres. Nucl Med Commun.

[28] Slart RH, Glauche J, Golestani R, Zeebregts CJ, Jansen JW, Dierckx RA (2012). PET and MRI for the evaluation of regional myocardial perfusion and wall thickening after myocardial infarction. Eur J Nucl Med Mol Imaging.

[29] Juarez-Orozco LE, Glauche J, Alexanderson E, Zeebregts CJ, Boersma HH, Glaudemans AW (2013). Myocardial perfusion reserve in spared myocardium: correlation with infarct size and left ventricular ejection fraction. Eur J Nucl Med Mol Imaging.

[30] Slart RH, Bax JJ, van Veldhuisen DJ, van der Wall EE, Dierckx RA, de Boer J (2006). Prediction of functional recovery after revascularization in patients with coronary artery disease and left ventricular dysfunction by gated FDG-PET. J Nucl Cardiol.

[31] Lautamaki R, Schuleri KH, Sasano T, Javadi MS, Youssef A, Merrill J (2009). Integration of infarct size, tissue perfusion, and metabolism by hybrid cardiac positron emission tomography/computed tomography: Evaluation in a porcine model of myocardial infarction. Circulation Cardiovasc Imaging.

[32] Cuculi F, Dall’Armellina E, Manlhiot C, De Caterina AR, Colyer S, Ferreira V (2014). Early change in invasive measures of microvascular function can predict myocardial recovery following PCI for ST-elevation myocardial infarction. Eur Heart J.

