# Supplementary material for: Early risk stratification using Rubidium-82 positron emission tomography in STEMI patients
Source: J Nucl Cardiol. 2017 Jul 17;26(2):471–82. doi: 10.1007/s12350-017-0993-x (PMC6430746; doi:10.1007/s12350-017-0993-x)
Supplement: Supplementary file 1 — Supplementary material 1 (PPTX 1535 kb) [file 12350_2017_993_MOESM1_ESM.pptx]

## Slide 1
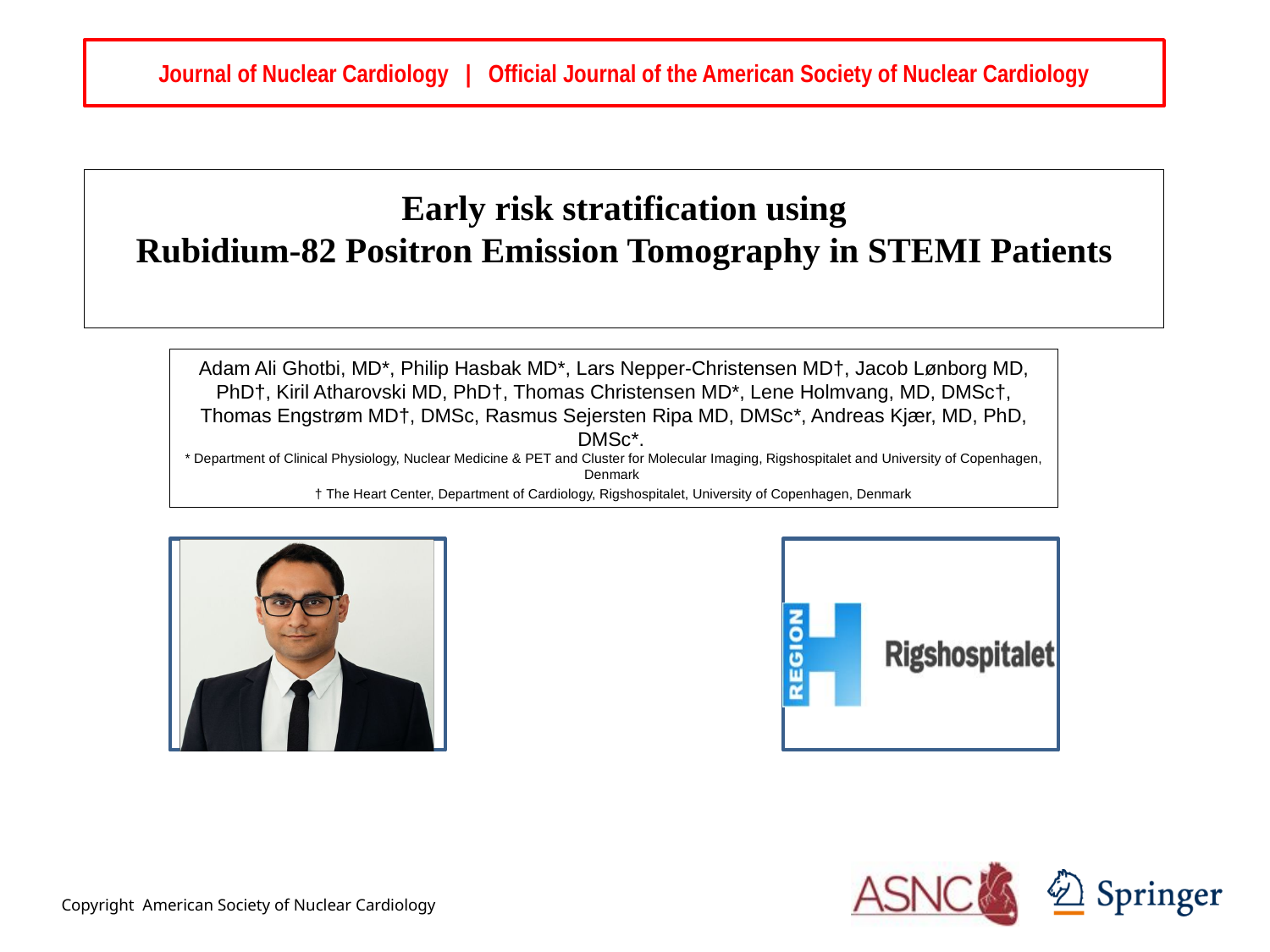

Journal of Nuclear Cardiology | Official Journal of the American Society of Nuclear Cardiology
# Early risk stratification using Rubidium-82 Positron Emission Tomography in STEMI Patients
Adam Ali Ghotbi, MD*, Philip Hasbak MD*, Lars Nepper-Christensen MD†, Jacob Lønborg MD, PhD†, Kiril Atharovski MD, PhD†, Thomas Christensen MD*, Lene Holmvang, MD, DMSc†, Thomas Engstrøm MD†, DMSc, Rasmus Sejersten Ripa MD, DMSc*, Andreas Kjær, MD, PhD, DMSc*.
* Department of Clinical Physiology, Nuclear Medicine & PET and Cluster for Molecular Imaging, Rigshospitalet and University of Copenhagen, Denmark
† The Heart Center, Department of Cardiology, Rigshospitalet, University of Copenhagen, Denmark
Head shot of author
required
Copyright American Society of Nuclear Cardiology

## Slide 2
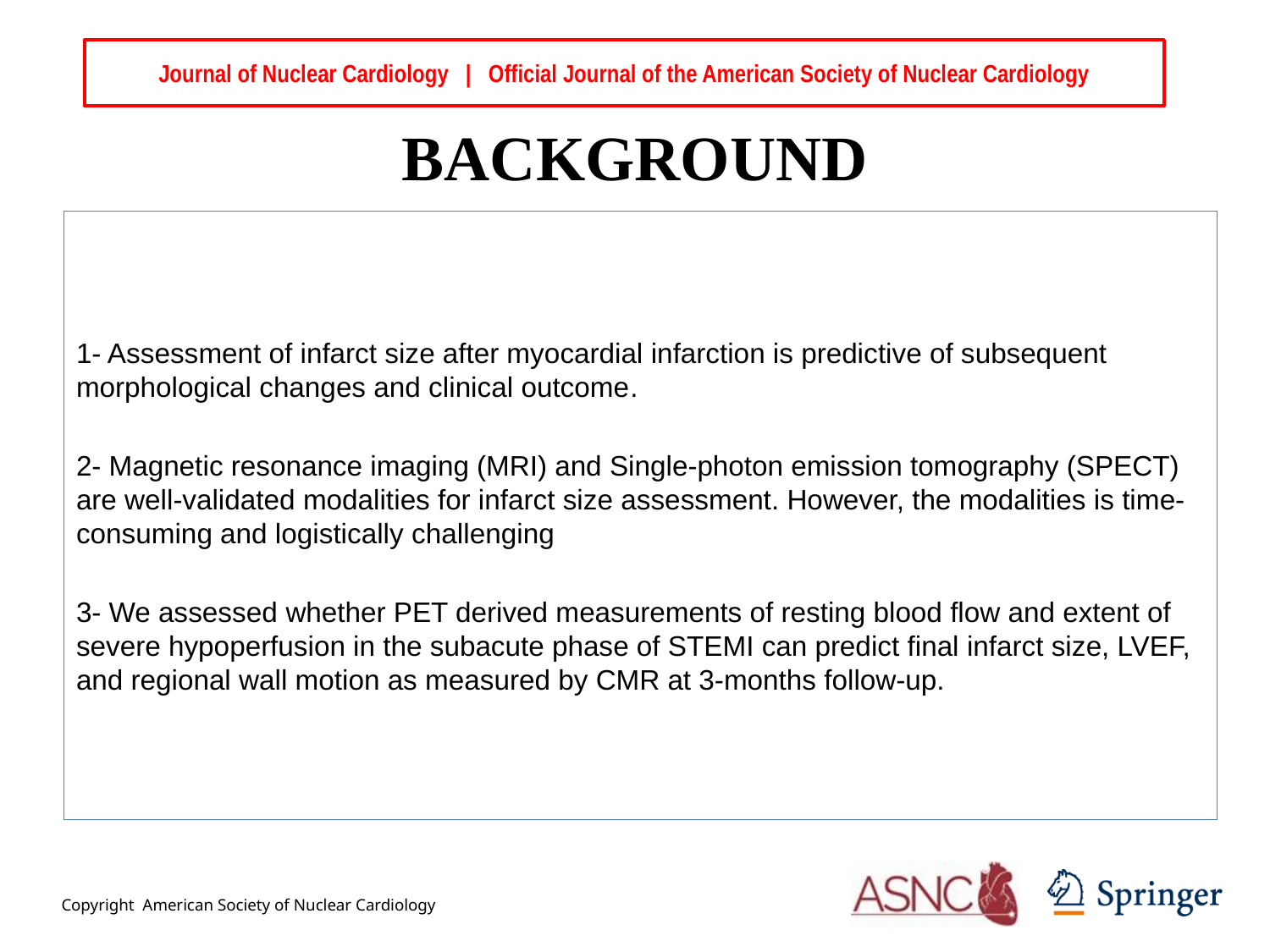

Journal of Nuclear Cardiology | Official Journal of the American Society of Nuclear Cardiology
# BACKGROUND
1- Assessment of infarct size after myocardial infarction is predictive of subsequent morphological changes and clinical outcome.
2- Magnetic resonance imaging (MRI) and Single-photon emission tomography (SPECT) are well-validated modalities for infarct size assessment. However, the modalities is time-consuming and logistically challenging
3- We assessed whether PET derived measurements of resting blood flow and extent of severe hypoperfusion in the subacute phase of STEMI can predict final infarct size, LVEF, and regional wall motion as measured by CMR at 3-months follow-up.
Copyright American Society of Nuclear Cardiology

## Slide 3
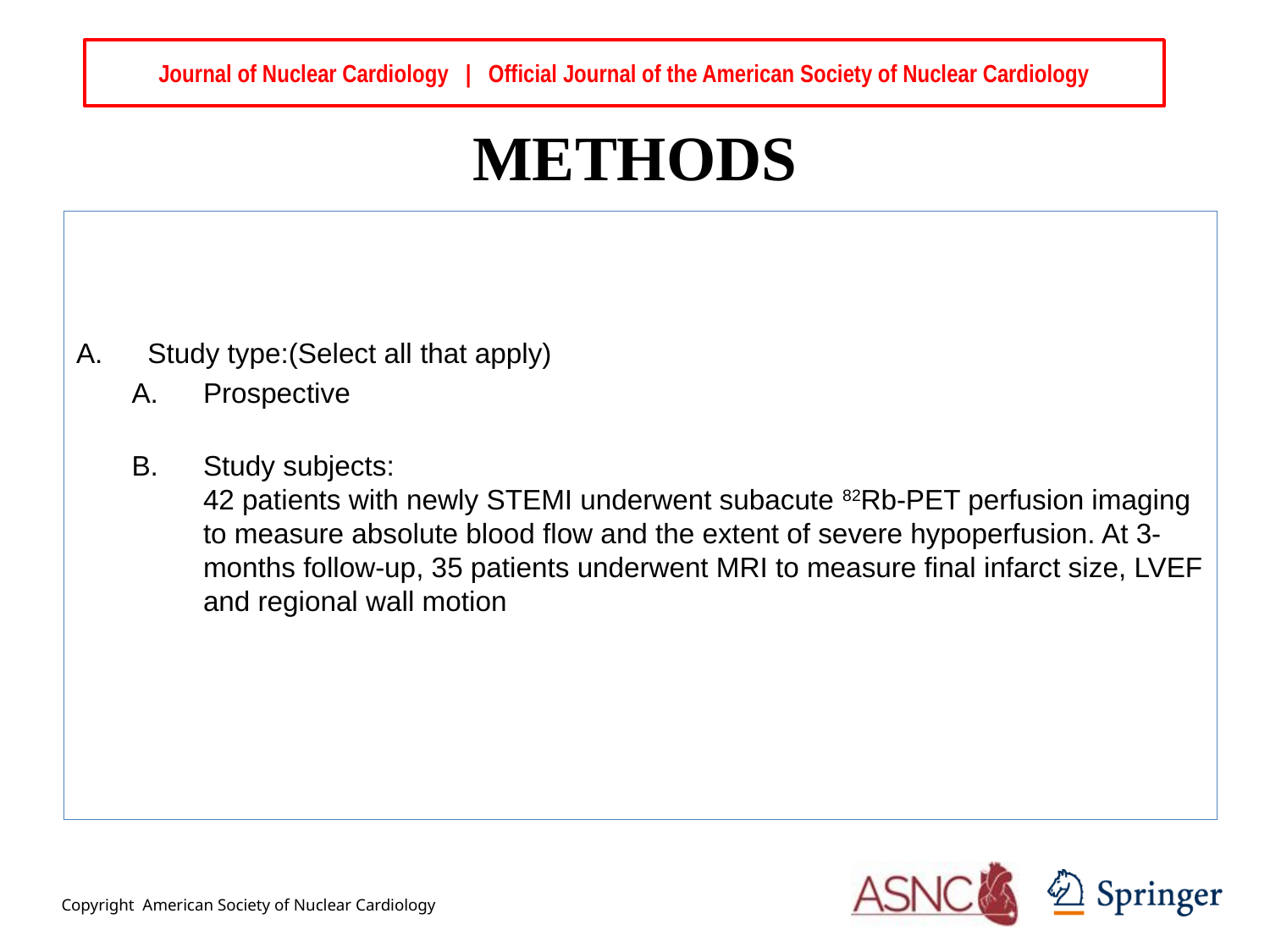

Journal of Nuclear Cardiology | Official Journal of the American Society of Nuclear Cardiology
# METHODS
Study type:(Select all that apply)
Prospective
Study subjects:
42 patients with newly STEMI underwent subacute 82Rb-PET perfusion imaging to measure absolute blood flow and the extent of severe hypoperfusion. At 3-months follow-up, 35 patients underwent MRI to measure final infarct size, LVEF and regional wall motion
Copyright American Society of Nuclear Cardiology

## Slide 4
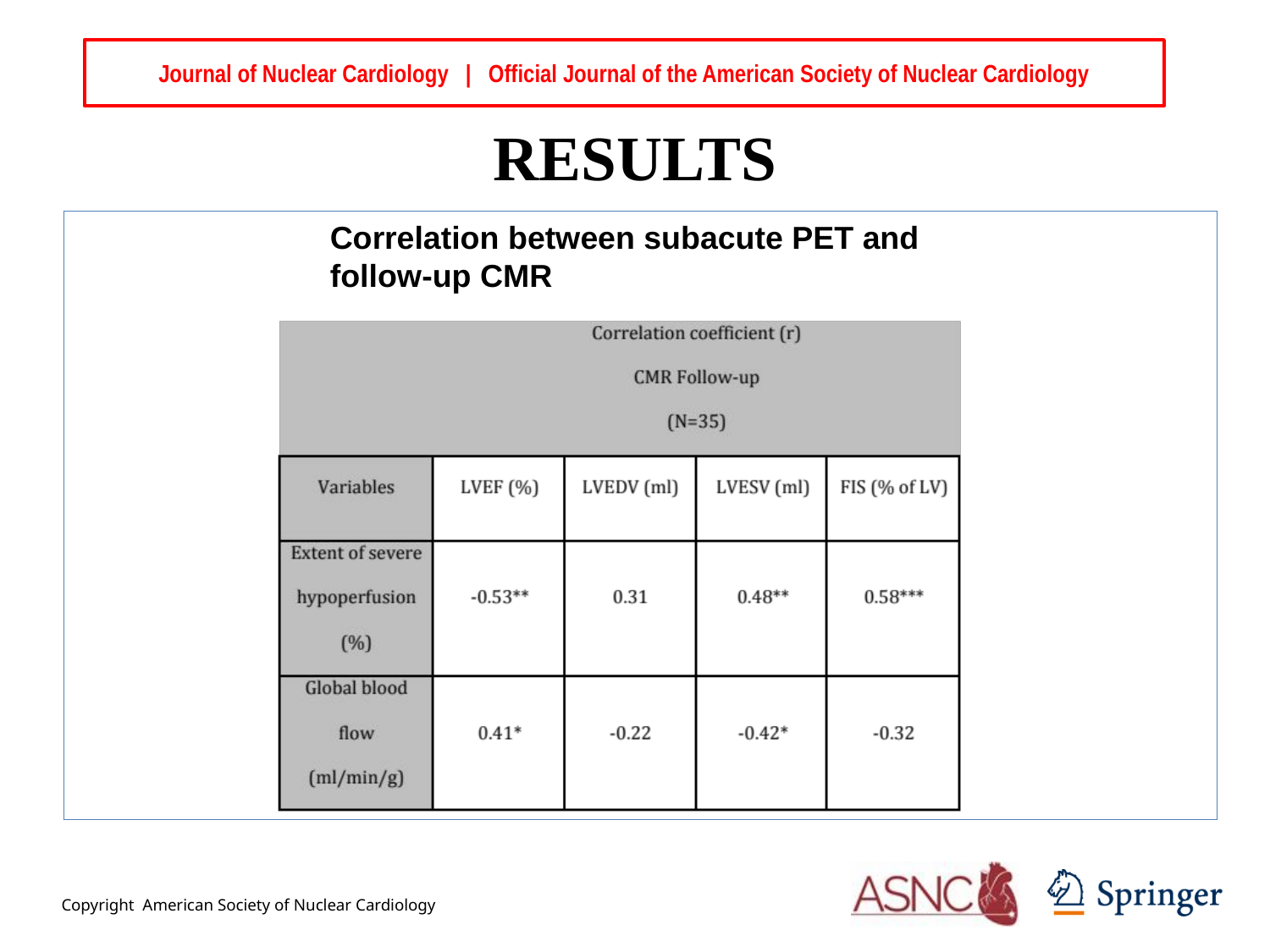

Journal of Nuclear Cardiology | Official Journal of the American Society of Nuclear Cardiology
# RESULTS
Correlation between subacute PET and follow-up CMR
Copyright American Society of Nuclear Cardiology

## Slide 5
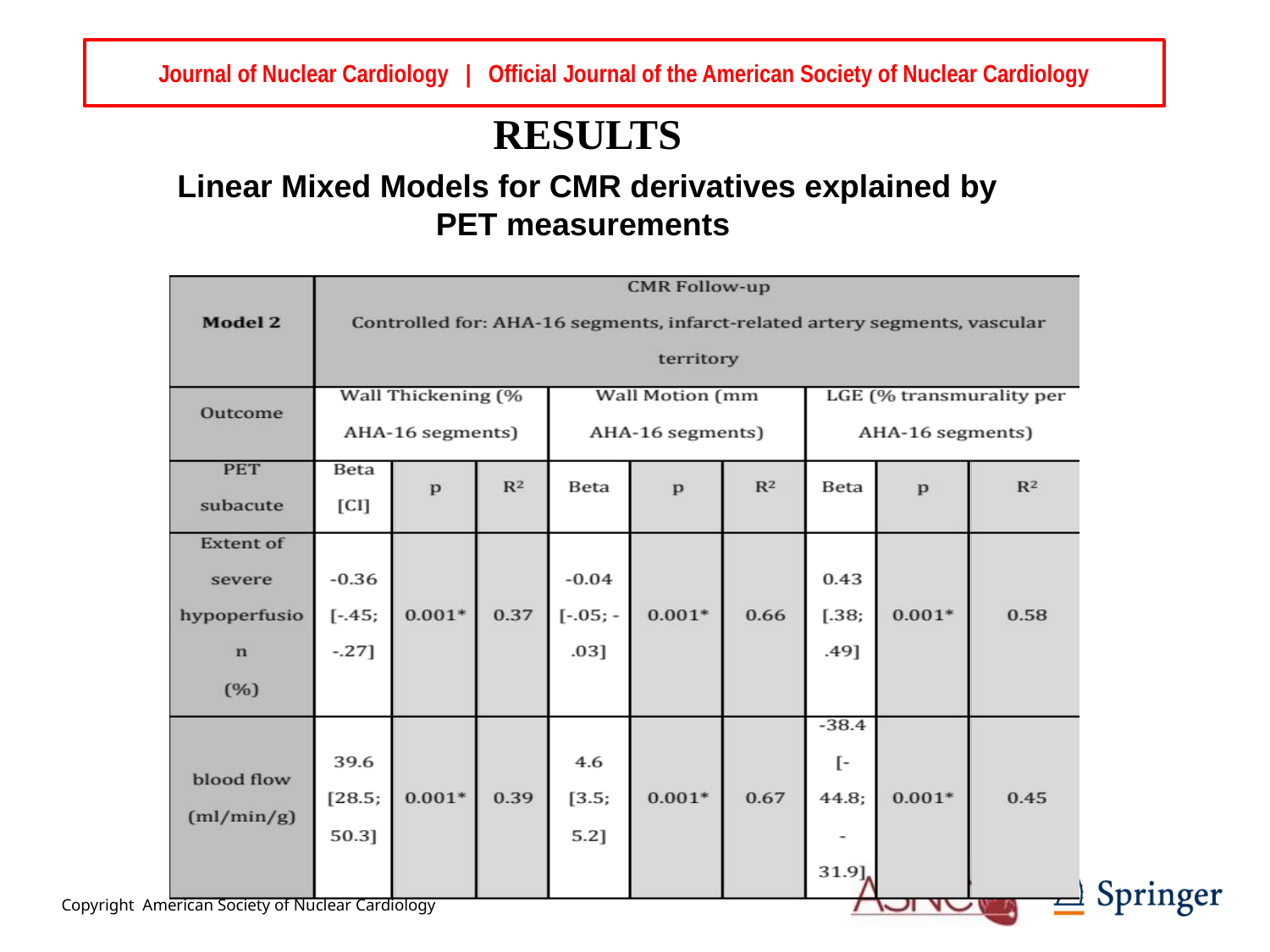

Journal of Nuclear Cardiology | Official Journal of the American Society of Nuclear Cardiology
# RESULTS
Linear Mixed Models for CMR derivatives explained by PET measurements
Copyright American Society of Nuclear Cardiology

## Slide 6
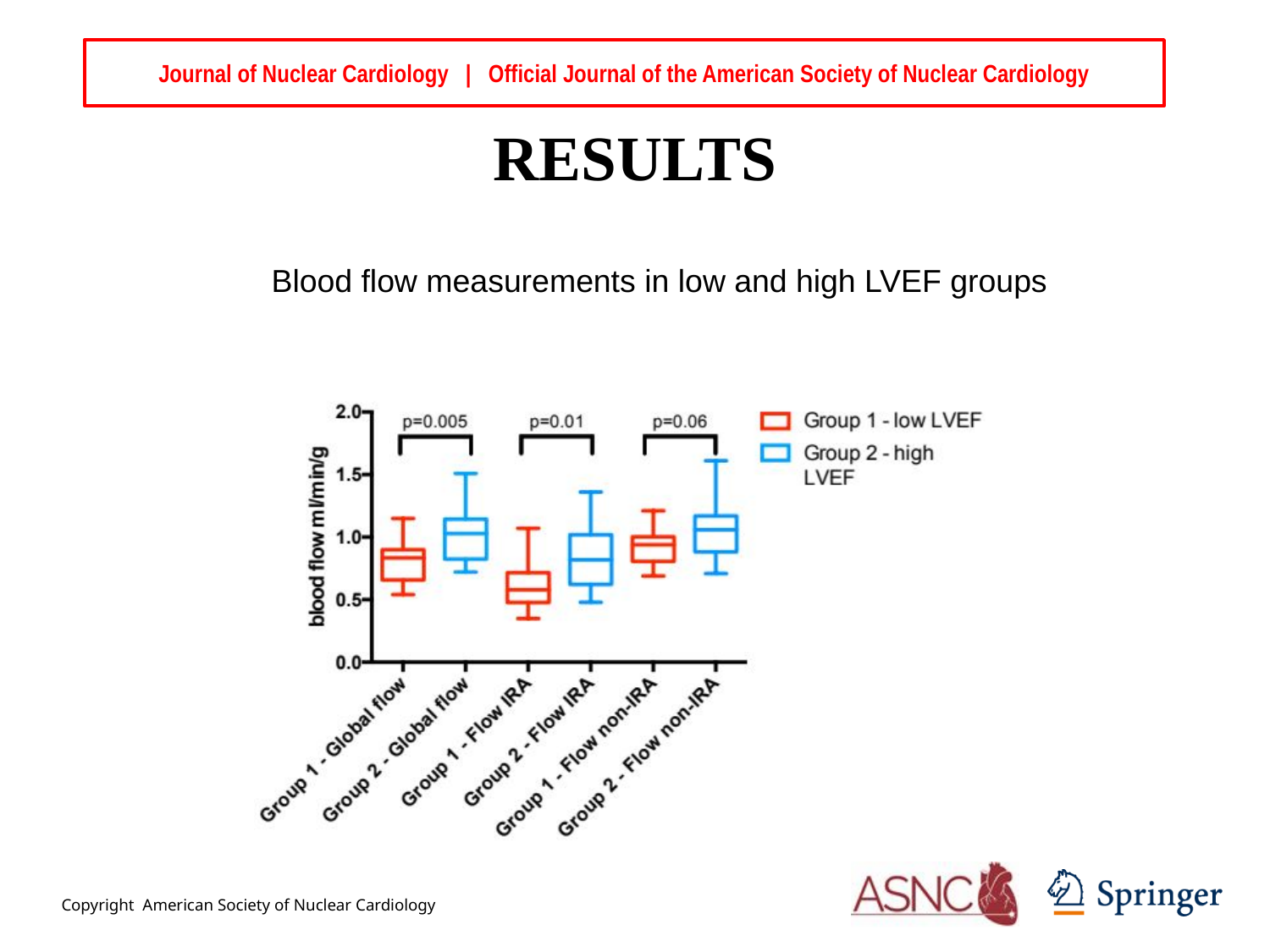

Journal of Nuclear Cardiology | Official Journal of the American Society of Nuclear Cardiology
# RESULTS
Blood flow measurements in low and high LVEF groups
Copyright American Society of Nuclear Cardiology

## Slide 7
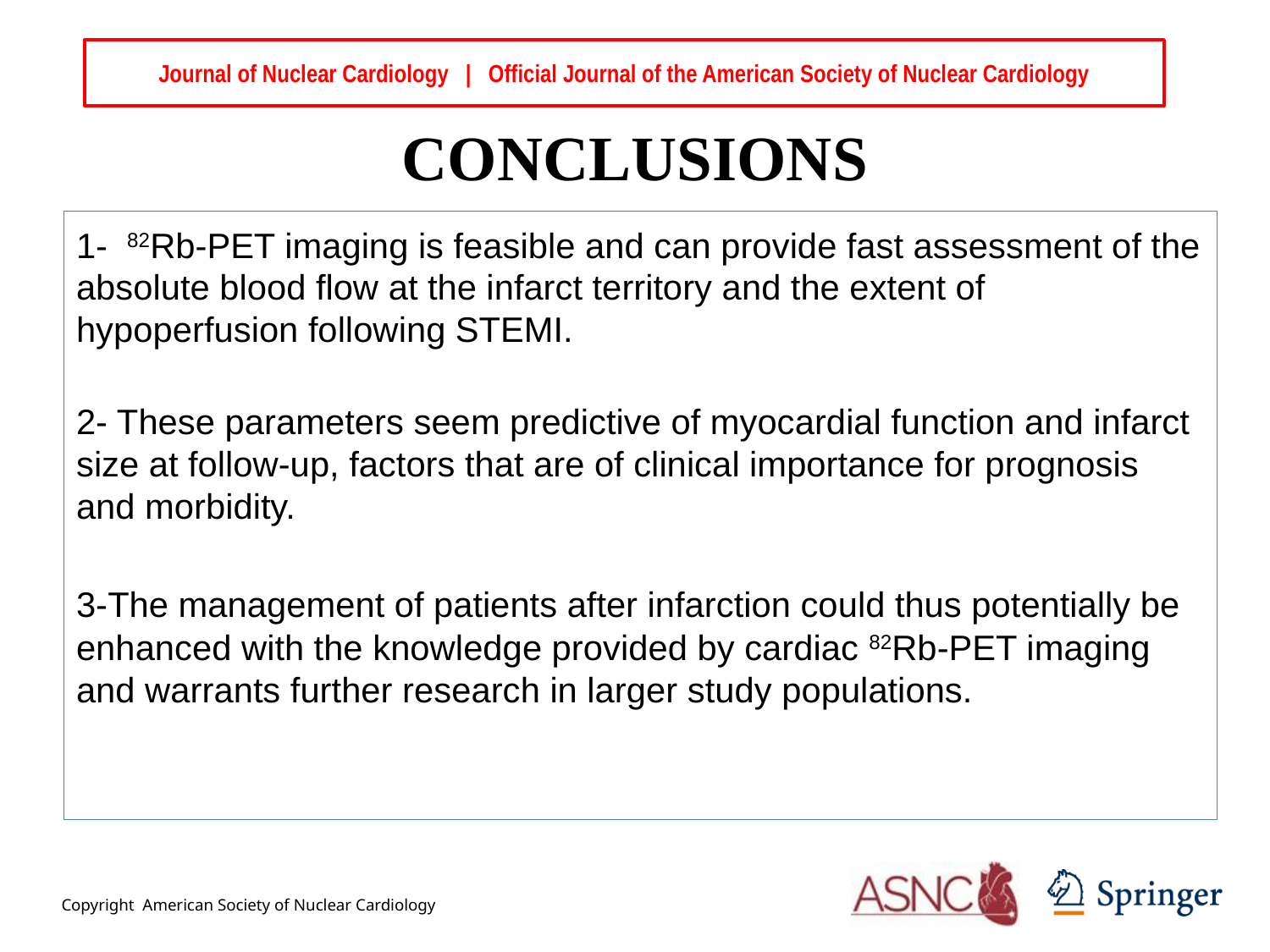

Journal of Nuclear Cardiology | Official Journal of the American Society of Nuclear Cardiology
# CONCLUSIONS
1- 82Rb-PET imaging is feasible and can provide fast assessment of the absolute blood flow at the infarct territory and the extent of hypoperfusion following STEMI.
2- These parameters seem predictive of myocardial function and infarct size at follow-up, factors that are of clinical importance for prognosis and morbidity.
3-The management of patients after infarction could thus potentially be enhanced with the knowledge provided by cardiac 82Rb-PET imaging and warrants further research in larger study populations.
Copyright American Society of Nuclear Cardiology
